# Electronic Records With Tablets at the Point of Care in an Internal Medicine Unit: Before-After Time Motion Study

**DOI:** 10.2196/30512

**Published:** 2022-02-10

**Authors:** Montserrat Pérez-Martí, Lina Casadó-Marín, Abraham Guillén-Villar

**Affiliations:** 1 Department of Information Technology Consorci Sanitari Alt Penedès-Garraf Vilafranca del Penedès Spain; 2 Department of Anthropology and Communication Universitat Rovira i Virgili Tarragona Spain; 3 Department of Nursing Universitat Rovira i Virgili Tarragona Spain; 4 Tarragona Spain

**Keywords:** electronic health records, nursing, computer handheld, equipment and supplies (devices tablets mobile phones, devices and technologies), workflow

## Abstract

**Background:**

There are many benefits of nursing professionals being able to consult electronic health records (EHRs) at the point of care. It promotes quality and patient security, communication, continuity of care, and time dedicated to records.

**Objective:**

The aim of this study was to evaluate whether making EHRs available at the point of care with tablets reduces nurses’ time spent on records compared with the current system. The analysis included sociodemographic and qualitative variables, time spent per patient, and work shift. This time difference can be used for direct patient care.

**Methods:**

A before-after time motion study was carried out in the internal medicine unit. There was a total of 130 observations of 2 hours to 3 hours in duration of complete patient records that were carried out at the beginning of the nurses' work shifts. We calculated the time dedicated to measuring vital signs, patient evaluation, and EHR recording. The main variable was time spent per patient.

**Results:**

The average time spent per patient (total time/patients admitted) was lower with the tablet group (mean 4.22, SD 0.14 minutes) than with the control group (mean 4.66, SD 0.12 minutes); there were statistically significant differences (W=3.20, *P*=.001) and a low effect (d=.44) between groups. The tablet group saved an average of 0.44 (SD 0.13) minutes per patient. Similar results were obtained for the afternoon shift, which saved an average of 0.60 (SD 0.15) minutes per patient (*t*_34_=3.82, *P*=.01) and high effect (d=.77). However, although there was a mean difference of 0.26 (SD 0.22) minutes per patient for the night shift, this was not statistically significant (*t*_29_=1.16, *P*=.25). The “nonparticipating” average age was higher (49.57, SD 2.92 years) compared with the “afternoon shift participants” and “night shift participants” (*P*=.007). “Nonparticipants” of the night shift had a worse perception of the project.

**Conclusions:**

This investigation determined that, with EHRs at the point of care, the time spent for registration by the nursing staff decreases, because of reduced movements and avoiding data transcription. It eliminates unnecessary work that does not add value, and therefore, care is improved. So, we think EHRs at the point of care should be the future or natural method for nursing to undertake. However, variables that could have a negative effect include age, night shift, and nurses’ perceptions. Therefore, it is proposed that training in the different work platforms and the participation of nurses are fundamental axes that any institution should consider before their implementation.

## Introduction

### Background

Nurses represent the category with the highest number of contracted professionals in the health care workforce and therefore the largest group of users of electronic health records (EHRs; in the countries where they are implemented) [1-3]. They use them as a primary tool for documenting, synthesizing, and communicating patient data. Therefore, introducing electronic devices into different work areas has a great impact on this group [2].

It is essential that the nurses are involved and committed to completing EHRs. Their use should be guided by nurses, as it is common for nursing professionals not to participate in their development [2,4-7]. EHRs should be usable for nurses and relevant for their practice [2,8].

Concepts such as usability, utility, efficiency in the context of the users’ use, and trust in technology are key elements for nurses to accept this innovation [8]. Therefore, we need to know the contributions or opportunities that they offer us, as well as their limitations.

Bibliography authors frequently highlight the opportunity offered by EHRs for developing new instruments that improve the quality and efficiency of care, such as standardized care plans, checklists, and decision support systems. Standardized care plans also prioritize the need to make the profession visible by offering results that demonstrate the effectiveness of care [2,5-7,9-13].

However, some authors state that they can act as a limiting agent for care, reducing critical thinking, clinical judgment, and basic nursing skills [5,10,14,15].

Another category that is important for the profession is saving time and communication. Some authors argue that EHRs decrease the time spent recording, because they have facilitating instruments such as copy-and-paste or drop-down menus with content standardization. They also promote access to information, thus improving inter- and multidisciplinary communication and consequently improving continuity of care [8-10,12,15].

Nevertheless, limiting agents in this category are also of equal importance. There is a greater volume of data available from any device, and finding information relevant to health care practice is not always easy. The user is forced to navigate the entire system, opening multiple screens; or using different software, with duplicated information, to obtain an overview of the patient's condition. The user is also required to enter a password repeatedly for the different programs, or the software has poor performance; there is an excess of mandatory information that needs to be entered; there are frequent interruptions; computers at the workstations are constantly busy, unavailable, or shared; or the setup has unfavorable ergonomics that deter nurses from trying to obtain information during the person's point of care. All these limiting agents describe a poor or poorly developed system that can cause interruptions in workflows in which the nurse has to perform more steps to carry out a nursing activity than would be necessary. More time spent on EHRs, time that adds no value, can lead to unnecessary delays in patient care. Another time-consuming cause that authors have found is a high percentage of transcription on paper of patient data, resulting in delays in patient information arriving to other professionals and increasing the possibility of error. This is experienced by nurses as work overload, a time limiter, and a barrier to communication. As a result, disruptions occur in the workflows, usability, and functionality of the program. It increases the time to record information and reduces the time of direct care to the patient [4,5,7,8,10-18].

When the right technology is successfully implemented, it can increase efficiency, decrease workloads, and provide time to perform direct care [19-21].

To improve these barriers, authors have proposed developing portable systems so that EHRs can be completed at the person’s point of care [13,18].

EHRs at the point of care promote patient quality and safety [13,19,21-25]. There are portable systems like reading barcodes for administering medication or to identify patients within the application as well as systems with early warning scores. These features increase patient safety. In addition, this system reduces errors from data transcription and latency time (information is recorded at the time it is obtained), which results in more accurate records. Quality is improved, and relying on remembering information is avoided. It provides accurate, real-time information, which improves accuracy and therefore patient safety. Resources that do not make it easy to complete EHRs at the patient’s bedside make it easier to make errors. For example, a health care professional cannot know if the biobiotic prophylaxis inserted by surgical intervention is correct if the electronic medical history is not registered [13,23-25].

Bedside patient EHRs also promote communication and continuity of care [8,14,22-26]. The possibility to access information within the room facilitates communication between professionals, with the patients, and with their families, making them part of their health process and thus increasing their satisfaction and continuity of care [8,14,23-27].

Furthermore, EHRs at the point of care improve workflows. They decrease travel time and avoid transcription, which save time as they reduce the time spent recording and increase the time spent with the patient [25-27].

However, authors have found that nurses themselves have varying perceptions and opinions about bedside patient EHRs. They are more satisfied with mobile devices and prefer to use them for complex patients (because they require a large amount of information) and to record vital signs and blood products [14,25].

However, they prefer not to use them in 2 specific situations: for noncomplicated patients, because their records are simple (require little data), and to admit the patient, because it takes a long time (it is better to find a quiet place to do so). Other reasons include that, while they are recording the information, they have to answer questions from patients and families (disrupting concentration), they feel that they are not giving good patient care (because they are concentrating on the screen), and they feel that it does not offer them the opportunity to disconnect (the post-record gives them time and space for this). In addition, bibliography authors also argue that documentation is not a high priority in the nurses’ activities, something that needs to be done straight away. Professionals do not feel that documentation affects the timeliness of patient care [17,24,27,28].

In other words, for nurses, bedside patient EHRs require mental and technical skills. However, they do not perceive that, if they do not complete them, it will impair the quality of the care provided.

The articles consulted refer to a variety of devices, and there is no consensus on the most appropriate resource for completing EHRs at the point of care. Evaluating this is complex and is related to many different factors in each center, such as the Wi-Fi connection, access, and identification system [22-26].

In the literature consulted, no scientific articles were found in Spanish states that evaluated the effectiveness of a practical experience with EHRs at the point of care.

From other information sources, there are 2 examples of practical experiences implemented in the Spanish state: the projects at the Hospital Infanta Cristina de Madrid and Osakidetza Hospital in the Basque Country [29,30].

All of these arguments show that it is important for nurses to decrease recording and technology time, as it is seen as a complementary, administrative, and bureaucratic task.

Nurses may perceive introducing technology as an increase in these bureaucratic tasks and a detriment to direct patient care. This situation may involve less physical contact with the patient and more time spent with electronic devices [14].

One of the nurse theories that can help understand this is Dr. Ray's Theory of Bureaucratic Care, which focuses on nursing in complex organizations, such as hospitals [31]. He explains that, if we rely only on administrative theories or on theories focused solely on the patient-nurse relationship, the organization will not be able to adapt to new needs. Economic benefits and competitiveness (bureaucracy) prevail in contemporary organizations. However, there has been a resurgence of nursing as an art of science focused on human care (patient-nurse relationship). The Bureaucratic Care Theory clarifies the meaning of human care in complex organizations, placing it at the center as it is an essential part of hospital management. Human care self-organizes, interrelates, and interconnects with each of its parts, placing spiritual-ethical care at the center (the engine that moves the nursing practice) and around it the bureaucratic factors, such as the educational, physical, sociocultural, legal, economic, political, and technological factors [31-33].

It is necessary to take into account all these arguments and reflections when implementing any changes to EHRs. We believe that completing EHRs at the point of care with tablets can meet nurses’ needs and expectations. It can strike a balance between nurses’ need to provide direct patient care and the requirement to complete EHRs, and thus improve and facilitate care.

### General Objective

The aim of this study was to evaluate whether completing EHRs at the point of care with tablets reduces nurses’ time spent on records compared with the current system. This time difference can be used for direct patient care.

### Specific Objectives

The first specific objective was to describe the sociodemographic variables (age and gender) of the nursing professionals in internal medicine units participating in the study, depending on the shift and the initial and final perceptions of the project.

The second was to describe and evaluate the sociodemographic variables (age and gender), shift, exclusion criteria, and initial and final perceptions of nurses working in internal medicine units who were not able to participate in the study.

The third was to compare the difference between the age groups of participants and those not participating in the study.

The fourth was to assess whether the implementation of this new record system decreases the time spent on patients compared with the current system and depending on the shift.

The results of this study were used to inform the impact of tablet use on workflows and to detect improvements to facilitate patient bedside registration.

## Methods

### Overview

A before-after single time motion study was conducted for 3 months (February 2017 to April 2017) in the internal medicine units of a regional hospital. A time and motion study is a quantitative data collection method in which the observer records the time and actions and movements of the participants. This type of study is often used for computer applications [34,35].

A total of 310 hours of observation and 130 observations of complete patient records were performed. Each observation could last between 2 hours and 3 hours and were carried out in the afternoon hours of 3:00 PM and 5:30 PM and during the night, from 10:00 PM to 1:00 AM (at the beginning of the nurses’ work shifts).

No sampling technique was applied. The sampling was of all the nurses working in the medical unit who met the inclusion criteria. A representative sample of all regional hospital nurses was ruled out because the working conditions did not allow a larger sample to be monitored. We therefore decided to focus on a single unit that would make research feasible in terms of time and resources. As the sample size was small, we decided to establish a minimum amount of observations for each participant in the control and experimental groups. The significance level was .05, and the beta risk was less than .2 in a bilateral contrast. The common standard deviation was 1.96, and therefore, the size would be 15 observations per control group and 15 observations for the experimental group to detect a difference ≥1.5 units, estimating a monitoring loss rate of 10%. These data were obtained with a sample size calculator [36].

Following these calculations, we decided to perform a minimum of 10 observations for each nurse, 5 for the control condition (current system) and 5 for the experimental condition (using the tablet). A total of 23 professionals could participate in this study for 3 months, as they were the only ones who had the minimum dedicated time to be able to be observed continuously; however, considering the inclusion criteria, there were 13 individuals who could participate satisfactorily. Each person could only be in 1 group.

The control group used a computer on wheels (current system), and the experimental group used the tablet.

The tools for gathering information were 2 ad hoc databases with the study variables.

### Inclusion Criteria

The inclusion criteria were as follows. The nurse was hired and worked in a stable job. A minimum of 10 observations could be made within 3 months, as this would facilitate the adaptation and learning curve in the experimental phase. The nurse carried out care activities in internal medicine units. This is because it is a robust unit, in which the occupancy rate is more stable and the average hospital stay is greater than 3 days. The nurse works the afternoon or night shift; the night shifts are longer than the other shifts because the staffing and distribution of activities are different [37]. The nurse volunteered to participate in the study. The nurse had a certain level of competence according to Benner’s skills acquisition model [38]: It is necessary that the nurse has worked more than 3 years in the hospital, because in this time, they can learn the functioning of the hospital and the EHRs as well as the protocols and procedures of the center [38]. The nurse can use information and communication technologies (ICT) [28].

### Demographic and Descriptive Variables

The variables age, sex, perception, and work shift of the participants and nonparticipants in the study were analyzed. The participants were those who were able to use the tablets according to the inclusion criteria: a total of 13. The nonparticipants were those who did not use the tablets: a total of 10.

Perception was measured by asking the professionals, at the beginning and at the end of the project, what their perception was: positive, neutral, negative.

There were 2 options for the shift variable (afternoon and night), according to the most common work shift of that nurse.

The exclusion criteria (only for nonparticipants) were measured using 5 items (sick leave, change of service, experience <3 years, does not accept, cannot use ICT).

### Principal Variables

The number of patients admitted was the total number of patients who were hospitalized at the time of observation. Although the medical unit has 14 beds available per nurse, only actual occupancy was measured.

The total time was the result of the sum of the ”round“ time and time to record the data in the EHR. A ”round“ time was defined as the routine established at the beginning of the afternoon and evening shifts when vital constants are taken, the nurse activities are standardized, and an overall assessment is made of the patients. There is no such routine in the morning.

The time spent per patient was obtained by dividing the total time by the number of patients admitted. In addition, it was identified as the main variable of the study, because it is more standardized.

### Control Variables

The following exceptional situations were defined: exitus, vital emergency, hospital discharge. In these cases, observations were discarded.

### Justification of Variables

The selection of variables was justified by a study conducted at a Toronto hospital that measured the time nurses spent completing EHRs. The variables studied were the total time, time spent per patient, and number of patients admitted. This study compared paper records versus electronic records [18].

Two studies have used the variables sex, age, nationality, and experience related to positive perception of EHRs [10,39].

A previous study ruled out observations in case of emergency or illness [22].

The rest of the variables were chosen to adapt the research to the study field, to the practical and real situation of the work units.

### Data Analysis

Statistical analysis was carried out with SPSS 17.00 (IBM Corp, Armonk, NY), and a significance level of *P*<.05 was applied. For categorical variables, a frequency calculation and contingency table were performed using likelihood ratio analysis (n<60). For the quantitative variables, for each of the groups, the Shapiro-Wilk normality test (n<30) was performed. The mean, SD, and variance were analyzed using an analysis of variance (ANOVA), and 2-factor averages for samples (control and experimental groups) were contrasted using the Student *t* test (Wilcoxon test when the sample was not normal). Following this analysis, when the results showed differences in averages between groups, Cohen d was applied to measure the distance or effect between the groups [40].

### Ethics Approval

This paper is part of the research for a doctoral thesis, with a favorable report from the Bellvitge Clinical Research Ethics Committee (reference PR191/16). To respect the privacy and confidentiality of the informants, the databases only worked with their coded names. Participants provided their verbal and written consent. Each participant was informed about the purpose of the study, the voluntariness to participate, and the right to leave at any time.

## Results

### Demographics and Qualitative Results

Of the 23 professionals stably working in the internal medicine unit, 13 (56%) were able to participate in the study, and 10 (43%) could not.

As we can see in Table 1, most nurses participating in the study were women (11/13, 84%) versus men (2/13, 15%). The average age was 38.08 (SD 1.40) years. The 2 groups were homogeneous in the variables studied; there were no statistically significant differences in age between sexes and shifts (sex Leven *P=*.04; Mann Whitney *P=*.37; shift Leven *P=*-.02; Mann Whitney *P=*.10).

**Table 1:** Demographics and qualitative results from participants and nonparticipants.

Variables		Participants (n=13), n (%)	Nonparticipants (n=10), n (%)
**Sex**			
	Male	2 (15)	1 (10)
	Female	11 (84)	9 (90)
**Age (years)**			
	<26	0 (0)	2 (20)
	26-30	1 (7)	1 (10)
	31-35	3 (23)	0 (0)
	36-40	4 (30)	1 (10)
	41-45	5 (38)	2 (20)
	46-50	0 (0)	1 (10)
	>50	0 (0)	3 (30)
**Initial perception**			
	Positive	6 (46)	6 (60)
	Negative	4 (30)	3 (30)
	Neutral	3 (23)	1 (10)
**Final perception**			
	Positive	9 (69)	N/A^a^
	Negative	1 (7)	N/A
	Neutral	3 (23)	N/A
**Shift**			
	Afternoon	7 (53)	5 (50)
	Night	6 (46)	5 (50)
**Exclusion criteria**			
	Work leave	N/A	2 (20)
	Change from the unit	N/A	1 (10)
	Experience <3 years	N/A	3 (30)
	No accept	N/A	2 (20)
	No ICT^b^ basic level	N/A	2 (20)

^a^N/A: not applicable.

^b^ICT: information and communication technologies.

The initial and final perceptions about the project after participating in the project did not vary statistically by shift or age variables. However, in the group of only women (n=11), the results varied because the women's final perceptions improved after they participated in the study. An initial positive perception was present for 45% (5/11), and 63% (7/11) had a final positive perception, with significant differences (verisimilitude ratio *P*=.03) with a substantial Cramer coefficient (v=.65).

Also, most nonparticipating nurses were women (9/10, 90%) versus men (1/10, 10%). The average age was 42 (SD 4.3) years. Table 1 details the variables for initial perception, shift, and exclusion criteria.

If we look at the relationship of these variables with the initial perception toward the project, we could see that there is homogeneity with age and that the relationship with the exclusion criteria could not be established because the data were not robust. However, a statistically significant relationship was obtained with the shift variable (verisimilitude ratio *P*=.04), and 80% (4/5) of the nonparticipants in the afternoon shift had a positive perception of the project, which was better than the perception of the night shift (2/5, 40%); this difference had a very strong coefficient of association (Cramer v, *P*=.97). After the study had been explained, nonparticipating subjects in the night shift had a worse perception or acceptance.

As shown in Multimedia Appendix 1 and Multimedia Appendix 2, statistically significant differences in age were evident between the group of nonparticipants (excluding the group with experience <3 years) and the group with the afternoon shift and night shift participants. The ”nonparticipating“ average age was 49.57 (SD 2.92) years compared with the average ages of 37.71 (SD 2.33) years for the “afternoon shift participants” and 38.50 (SD 1.60) years for the “night shift participants” (Tukey afternoon *P*=.007; Tukey night *P*=.01).

### Principal Findings

The quantitative variables were used to measure and compare the times required to carry out the “round” and EHRs between the control group (current system) and the experimental group (tablet).

Of the total sample, the mean total time obtained for the control group was 55.44 (SD 2.11) minutes, and there was an average 11.77 (SD 0.25) patients admitted. For the tablet group, the average total time was 48.30 (SD 2.24) minutes, and there was an average 11.37 (SD 0.28) patients admitted.

Comparing the time spent per patient (the main variable of the study), it was evident that the average time spent per patient was lower with the tablet group mean 4.22, SD 0.14 minutes) than with the control group (mean 4.66, SD 0.12 minutes); there was a statistically significant difference (W=3.20; *P*=.001) and a low effect (d=.44) between groups (Table 2).

**Table 2:** Comparison of the average time spent per patient for the entire sample.

Variable	Control		Tablet		Comparisons			
	Mean (SD)	SW^a^	Mean (SD)	SW	Mean difference (SD)	W test^b^	*P* value	Cohen d
Time spent per patient (minutes)	4.66 (0.12)	.07	4.22 (0.14)	.006	0.44 (0.13)	–3.20	.001	.44

^a^Shapiro Wilk *P*<.05.

^b^Wilcoxon.

However, if we focused on analyzing these variables while taking into account the shift, the results brought a nuance or specificity to these more general data. The mean number of patients admitted was homogeneous and similar in the control (12.63, SD 0.22 patients) and tablet (12.50, SD 0.39 patients) groups. There were no statistically significant differences in their distribution (W afternoon *P*=.22; W night *P*=.96).

The average total afternoon shift times were 44.83 (SD 2.21) minutes for the control group and 35.48 (SD 1.17) minutes for the tablet group. The control group's night shift lasted 67.83 (SD 2.22) minutes, and the tablet group’s night shift lasted 63.27 (SD 2.78) minutes. The comparison of the average factor for related samples showed that there were significant differences in the afternoon shift (*t*_34_= 4.07, *P*<.001), with a high effect between groups (d=.93), but not in the night shift (*t*_29_=1.29, *P*=.20).

In the afternoon shift for the control group, the average times spent per patient were 4.07 (SD 0.13) minutes in the control group and 3.47 (SD 0.10) minutes in the tablet group. In the night shift, the control group spent an average 5.36 (SD 0.13) minutes, and the tablet group spent an average 5.09 (SD 0.19) minutes. Comparison of the average factor for related samples showed that, in the afternoon shift, the mean time spent per patient was lower with the tablet group, with a statistically significant difference (*t*_34_=3.82, *P*=.01), and there was a high effect (d=.77) between groups. However, the same results were not obtained in the night shift (*t*_29_=1.16, *P*=.25; Table 3 and Table 4).

**Table 3:** Average comparisons of total time, number of patients admitted, and time spent per patient for the afternoon shift.

Variable	Control		Tablet		Average comparison			
	Mean (SD)	SW^a^	Mean (SD)	SW	Mean difference (SD)	Test	*P* value	Cohen d
Total time (minutes)	44.83 (2.21)	.055	35.48 (1.17)	.47	9.37 (2.30)	4.07^b^	.001	.93
Number of patients admitted	11.03 (0.38)	.03	10.40 (0.33)	.24	.62 (0.49)	–1.20^c^	.22	—^d^
Time spent per patient (minutes)	4.07 (.13)	.21	3.47 (.10)	.25	.60 (0.15)	3.82^b^	.01	.77

^a^Shapiro Wilk *P*<.05.

^b^Student *t* test.

^c^Wilcoxon.

^d^Not calculated.

**Table 4:** Average comparisons of total time, number of patients admitted, and time spent per patient for the night shift.

Variable	Control		Tablet		Average comparison		
	Mean (SD)	SW^a^	Mean (SD)	SW	Mean difference (SD)	Test	*P* value
Total time (minutes)	67.83 (2.22)	.38	63.27 (2.78)	.85	4.56 (3.52)	1.29^b^	.20
Number of patients admitted	12.63 (0.22)	.01	12.50 (0.39)	.00	0.13 (0.49)	–.04^c^	.96
Time spent per patient (minutes)	5.36 (0.13)	.16	5.09 (0.19)	.37	0.26 (0.22)	1.16^b^	.25

^a^Shapiro Wilk *P*<.05.

^b^Student *t*.

^c^Wilcoxon

## Discussion

### Principal Findings

The results confirm the overall objective of this study: Completing EHRs at the bedside with tablets reduces nurses’ time spent recording compared with the current system in which nurses use a computer on wheels. This difference is due to improved workflows, as bedside EHR completion with a tablet avoids unnecessary travel, facilitates access to information, and avoids duplicating the work involved in data transcription. The same results were obtained for the afternoon shift but not for the night shift. Therefore, this registration system can meet the expectations of nurses and produce a positive impact on work dynamics since it covers 2 important needs for these professionals: saving time on bureaucratic tasks and having more time for care.

However, the perceptions of the participants and nonparticipants in the study did not always coincide with this premise. There was no initial broadly positive perception of this registration system. The nonparticipants were older than the participants, and of these, nonparticipants on the night shift had a worse perception toward the project.

### Comparison With Prior Work

The literature consulted shows conflicting results regarding whether electronic records reduce the time spent on records by citing numerous benefits and limiting agents. However, there is consensus on the concept for time: For nurses, time is an important and present concept. During their working day, they carry out numerous activities, always keeping in mind the way of organizing these so they can do them all in their work shift. Nursing is a pragmatic profession, in which an activity has to have a certain result. However, it is also a profession involving contact and a relationship with the patient through providing care, which is the main axis and motivation of their profession. Therefore, saving time in bureaucratic activities, to have more time for human care, is a constant concern [2,4,7-15,33,34,37].

There are other similar studies that investigated the impact of EHRs in workflows. They argue that, even though EHRs have been implemented, paper and subsequent transcription of information are still used. There is unanimity in saying that these practices are not advisable but differ in the problems they can cause, such as increasing latency and transcription errors [18,19] and duplication of work process [41].

Other authors who have published articles related to EHRs at the point of care have reached similar conclusions. It takes less time to enter the records because there is no need to move to a different place to enter the record, and work is halved because there is no need to enter handwritten records in the computer [20,22-24].

It is difficult to make comparisons between this study and other similar studies in which the time spent on EHRs at the point of care was calculated and demonstrated that technology can reduce time in registration or administrative tasks by nurses [19,20] due to the use of different research methods, variables, and resources. In the first article [19], the authors found a 30% reduction in time spent on records. The second [20] article obtained an increase of 6% in time devoted to direct care and a reduction of 12% time in administrative tasks. In this investigation, time saved using the tablets was 0.44 (SD 0.13) minutes; for the afternoon shift, it was 0.60 (SD 0.15) minutes. In the investigation by Wong et al [19], differences between hospitalization units and the night shift were not considered due to the observations occurring from 9 AM to 5 PM. In our investigation, we found differences between shifts. In 2 other studies [19,20], they compared paper versus EHRs at the point of care, and in our study, we compared EHRs that were not performed next to the patient versus EHRs at the point of care.

In the literature that we consulted, 3 studies studied the relationship between sociodemographic variables and the impact and acceptance by nurses of information systems. However, they were studied to achieve different objectives and with different results, but similar conclusions could be reached. One study found consistent data related to previous experience in the use of computers with more favorable attitudes toward EHRs [10]. Another investigation revealed that performance expectancy and social influence were significant predictors of nursing information system usage intentions and suggested that nursing managers should promote usage [39]. The third research study obtained more negative results and claimed that the use of mobile devices intensifies the negative effects of usability problems related to EHRs, and they suggested different actions related to improving the usability and interface of the applications. Moreover, they referred to the relationship that the nurse's experience has with pressure and distress [1]. According to our results, the initial perception of the nurses was not unanimously positive, and the findings related a worst perception with age and the night shift. We do not know if age may be related to inexperience in the use of ICT, the interface, or fear of change or the unknown. Moreover, it was not possible to determine the factors related to the shift.

In order to improve acceptance, we agree with other authors [1,3,10,39], that nursing management should promote bedside EHRs and explain their benefits but should also offer continuing education courses and sufficient training in information systems to all nurses. Resources should be invested to improve the interface through the participation of nurses in its development.

### Study Limitations

The time the researchers dedicated to the study was limited because it was carried out outside of working hours, there were few resources available to carry out the research (one tablet), and there were huge difficulties in making 10 observations with the same participant. For these reasons, there are limitations in terms of sample size and nonprobabilistic sampling type. To ensure external validity, this research could be repeated with a larger sample and random sampling in the future.

However, no greater control was taken over the confusion variables that could cause the results to vary. This would improve internal validity.

Finally, the number of nursing activities recorded per patient (eg, vital constants, pain, catheter, oxygen therapy, health education, scales) was not quantified at the time of observations. This was to avoid the effect of the observer and to promote informal acceptance of the study by the participants. The cost benefit of implementing these measures would have to be assessed for future studies.

### Conclusions

This investigation allows us to know the impact EHRs at the point of care can have on workflows.

First, our findings determined that, with bedside EHRs, the time spent in registration by the nursing staff decreases, because of reduced movements and elimination of data transcription. Because EHR completion at the bedside eliminates unnecessary work that does not add value, care is improved. So, we think EHRs at the point of care should be the future or natural method for nursing to undertake.

On the other hand, our study explored sociodemographic and qualitative variables associated with this new registration system. It allows us to identify the factors that can make people reluctant to participate in a technological project, and we performed actions aimed at solving them in order to anticipate the possible obstacles. Otherwise, we could make statements that are wrong or biased and do not correspond with reality nor solve the problem. However, more studies with a larger sample would be needed to improve the validity of these results. It is proposed that training in the different work platforms and the participation of nurses are fundamental axes that any institution should consider.

This research is the result of the preparation of a doctoral thesis, and these findings will be triangulated in the second part when a qualitative phenomenological study is conducted on the experience and perceptions of nurses with EHRs at the point of care.

Taking into account that the subject of this research is quite unknown, especially in the Spanish territory; daily use of technology is part of our society; and nurses are the capital of hospital work templates, it is imperative to go in-depth in similar studies to provide more information and allow us to develop systems that promote patient care.

## Appendix

Multimedia Appendix 1 Average analysis of variance (ANOVA) comparison of the age variable.

Multimedia Appendix 2 Multiple comparisons of averages between the groups for the age variable.

## Abbreviations

ANOVA: analysis of variance
EHR: electronic health record
ICT: information and communication technologies
