# Supplementary material for: Electronic Records With Tablets at the Point of Care in an Internal Medicine Unit: Before-After Time Motion Study
Source: JMIR Hum Factors. 2022 Feb 10;9(1):e30512. doi: 10.2196/30512 (PMC8874839; doi:10.2196/30512)
Supplement: Multimedia Appendix 1 [file humanfactors_v9i1e30512_app1.doc]

Appendix 1: Table A1. Average comparison (ANOVA) of age variable.

| Variable | | Mean (SD) | Range | SW^a^ | Leven Test | *P* value | F test^b^ | *P* value |
| --- | --- | --- | --- | --- | --- | --- | --- | --- |
| **Age (years)** | |  |  |  |  |  |  |  |
|  | Afternoon shift participants | 37.71 (2.33) | 36-40 | .53 | 2.80 | .89 | 7.69 | .004 |
|  | Night shift participants | 38.50 (1.60) | 36-40 | .051 | 2.80 | .89 | 7.69 | .004 |
|  | Non- participants^c^ | 49.57 (2.92) | 46-50 | .41 | 2.80 | .89 | 7.69 | .004 |

^a^Shapiro Wilk *P*<.05

^b^F Snedecor *P*<.05

^c^Excluded <3 years of experience
