# Supplementary material for: Electronic Records With Tablets at the Point of Care in an Internal Medicine Unit: Before-After Time Motion Study
Source: JMIR Hum Factors. 2022 Feb 10;9(1):e30512. doi: 10.2196/30512 (PMC8874839; doi:10.2196/30512)
Supplement: Multimedia Appendix 2 [file humanfactors_v9i1e30512_app2.doc]

Appendix 2: Table A2**.** Multiple comparisons of average between groups, age variable.

| Variable | | Difference mean (SD) | *P* value^a^ |
| --- | --- | --- | --- |
| **Age (years) non-participants^b^** | |  |  |
|  | Afternoon shift participants | 11.85 (3.34) | .007 |
|  | Night shift participants | 11.07 (3.48) | .01 |

^a^Turkey *P*<.05

^b^Excluded <3 years of experience
